# Incidence and risk factors of prolonged recovery during procedural sedation in pediatrics

**DOI:** 10.3389/fmed.2024.1466205

**Published:** 2024-10-11

**Authors:** Yu Cui, Qunying Wu, Min Tang, Qin Chen, Lu Kang, Qixia Mu, Yani He

**Affiliations:** Department of Anesthesiology, The Affiliated Hospital, School of Medicine, UESTC Chengdu Women’s & Children’s Central Hospital, Chengdu, China

**Keywords:** prolonged recovery, procedural sedation, pediatric patients, risk factors, incidence

## Abstract

**Background:**

Sedation-related adverse events not only referred to a cause for morbidity and mortality but also included events that could disrupt routine procedures and thus lead to reduced procedural efficiency or quality. To date, no literature is available to predict the risk factors associated with prolonged recovery in pediatric patients during procedural sedation. Thus, we retrospectively analyzed the two-year sedation data to explore the above questions.

**Methods:**

Pediatric patients who underwent procedural sedation between January 2022 and January 2024 were retrospectively analyzed. The patients were divided into two groups according to sedation duration <120 min (Non-prolonged recovery group); or ≧ 120 min (Prolonged recovery group). The primary outcome was the incidence of prolonged recovery. Risk factors associated with prolonged recovery were assessed.

**Results:**

A total of 30,003 patients were identified; 854 (2.8%) developed prolonged recovery during procedural sedation. By multivariate regression, a higher body weight (OR 1.03, 95%CI 1.01–1.05), outpatients (OR 1.31, 95%CI 1.07–1.59), patients with sedation history (OR 1.25, 95%CI 1.07–1.44), and patients received chloral hydrate (OR 1.47, 95%CI 1.06–2.03), were associated with increased odds of the prolonged recovery with the initial sedative(s).

**Conclusion:**

Monitoring time needs to be extended in patients with sedation history, those with heavier weights, outpatients, and those who received chloral hydrate.

## Background

To obtain high-quality images, most diagnostic examinations require patients to remain stationary. However, for pediatric patients, it is difficult to actively cooperate with this requirement. Thus, sedatives are often prescribed to them when they intend to undergo diagnostic examinations, such as computed tomography (CT), magnetic resonance imaging (MRI), and echocardiogram.

Sedation-related adverse events were well-reported by a review (1). The review pointed out that sedation-related adverse events not only referred to a cause for morbidity and mortality, but also included events that could disrupt routine procedures and thus lead to reduced procedural efficiency or quality (1). From a resource utilization perspective, the sedation duration should be followed with interest since the patients who have been sedated must be monitored closely until they are fully recovered. Prolonged recovery is one of the commonly seen side effects during procedural sedation (2–5), which may increase the time consumption for both healthcare staffs and guardians, enhance the workload of staff, and reduce parental satisfaction. Besides, patients experiencing prolonged recovery were at risk of developing adverse events, such as long fasting time induced hypoglycemia and hypovolemia, and potential airway issues (6). A systematic review reported that the incidence of prolonged recovery during procedural sedation ranged from 0.18% ~ 30% (5). The occurrence of prolonged recovery resulted from pharmaceutical effects, metabolic conditions, and patient-related factors (2, 5). In clinical practice, chloral hydrate, dexmedetomidine, midazolam, melatonin, and thiopental could be selected for procedural sedation, either individually or in combination (5). Considering both safety and efficacy, the optimal medication or combination of medications is yet to be determined. Theoretically, a drug with a high potency could result in an acceptable sedation success rate, but the likelihood of adverse events (i.e., prolonged recovery) occurring might also increase accordingly.

Therefore, identifying the underlying causes of prolonged recovery after procedural sedation with the initial dose is beneficial to developing appropriate sedation strategies, accelerating the patient’s recovery, and increasing parental satisfaction. However, to date, no literature is available to predict the risk factors associated with prolonged recovery in pediatric patients during procedural sedation. Thus, we retrospectively analyzed the 2 years of sedation data to explore the above questions.

## Methods

We performed a retrospective, case–control study which was conducted at Chengdu Women’s and Children’s Central Hospital. Ethical approval was obtained from Institutional Review Board (IRB) of our institution [Approval number 2024(37)]. In our center, chloral hydrate was the most commonly used drug during non-invasive diagnostic procedures until 2022; whereas, after 2022, dexmedetomidine and midazolam were used increasingly. To understand the properties of the different drugs, patient data from January 2022 to January 2024 was extracted from electronic medical record system. A total of 30,003 cases were identified. The need for informed consent was waived because of the use of anonymous patient data. Age groups were defined as follows: neonates (1 day–1 month), infants (1–12 months), toddlers (1–3 years), and older children (> 3 years), which was in line with our previous work (7).

### Patient selection

*Inclusion criteria*

- Patients who received sedative medication(s) for non-painful diagnostic procedures
- Patients were well-sedated and completed the diagnostic procedures by only accepted initial dose of sedative(s). The initial dose of sedative(s) referred to that the patient can complete the procedure with only one dose of medication (which could be a single drug or a combination of drugs). Patients who are well-sedated are those who have completed the diagnostic procedure and obtained acceptable images.

*Exclusion criteria*

- The awake time of patients was not well-documented.
- The rarely used medication regimens, such as only 1 case and 2 cases received mida+ketamine or chloral hydrate+propofol as the initial sedative, respectively.
- Patients were unconscious, lethargic, or unresponsive before sedation.
- Patients with severe respiratory obstruction or severe cardiac arrhythmia before sedation.
- Patients could not achieve a target depth of sedation with the initial type and dose of medication.
- Patients were assisted by mechanical ventilation before sedation.

### Sedation procedure

In our institution, patients are referred to sedation center when they have to receive sedative medications for diagnostic procedures, and about 20,000 patients are sedated per year. The sedation regimen is made by the attending anesthesiologist according to the nature of the diagnostic procedures and the characteristics of the child, which is implemented by the nurses. Heart rate and pulse oxygen saturation are monitored and recorded. Attending anesthesiologists with more than 2 years of experience in pediatric anesthesia manage patients in the sedation center. If adverse events occur, the attending anesthesiologist on duty takes responsibility for handling them.

### Data collection and definitions

Patients gender, age, weight, sedation history, outpatients/inpatients, sleep deprivation, type of procedures, type of initial sedatives, and sedation duration were collected. When the patient is fully awake after sedation, they will discharge from the sedation center. The sedation recovery time is defined as the period between administration of sedative(s) until the patient is totally awake and alert and discharged from the sedation center. Totally awake is defined as the patients can open their eyes, have purposeful movements, or cry. Due to repeated administration of sedatives may lead to an extension in sedation duration, only patients who accept initial dose of sedative medication are included. Moreover, the duration of sedation cannot be calculated if patients fail to be sedated; thus, patients who achieve the target depth of sedation and complete the diagnostic procedures will be analyzed in the final analysis. Prolonged recovery is defined as the duration of sedation ≥120 min, which bases on the publication by Zhou et al. (3). Thus, in the current study, the patients were divided into two groups according to sedation duration as follows. If the patients have stable vital signs, no special management measures will be given by the sedation providers. In our routine practice, we rarely prescribe drugs to accelerate awakening during the awakening period, expect the sedation duration to exceed 240 min.

- Non-prolonged recovery group (NPSR group): the sedation recovery time < 120 min
- Prolonged recovery group (PSR group): the sedation recovery time ≧ 120 min

### Outcomes

The primary outcome is the incidence of prolonged recovery. Risk factors associated with prolonged sedation recovery time are assessed as secondary outcomes.

### Statistical analysis

Statistical analysis was conducted with R Studio, version 4.2.2. Categorical variables were presented as numbers and percentages (%). χ2 or Fisher exact tests were used as appropriate. Continuous variables were presented as the mean ± standard deviation (SD) if normally distributed, and Student’s *t*-tests were used for comparison between the two groups; otherwise, data was reported as medians and interquartile ranges (IQR) (25–75%), and the Mann–Whitney U test was used for comparisons. One-way ANOVAs were used for multiple comparisons. *p* values <0.05 were considered statistically significant.

A univariate regression analysis was performed to identify the potential candidate predictors associated with prolonged sedation recovery at the initial drug(s). Variables with significance levels of *p* ≤ 0.1 in the univariate analysis were added to the further multivariate regression model, as described by Gosling et al. (8) and Cui et al. (9). Least Absolute Shrinkage and Selection Operator (LASSO) regression was further conducted with the ‘glmnet’ function in R to identify the most relevant predictors as multivariate regression analysis. Lambda_1se = 0.002380073 was selected and used to refit the model, which resulted in a stricter penalty that allowed us to reduce the number of covariates even further than with the former Lambda (10). Factors with non-zero coefficients were selected. The multicollinearity of the development models was evaluated by variance-inflation factors (VIF). When VIF was greater than 5, significant multicollinearity was considered.

## Results

**Figure 1:**
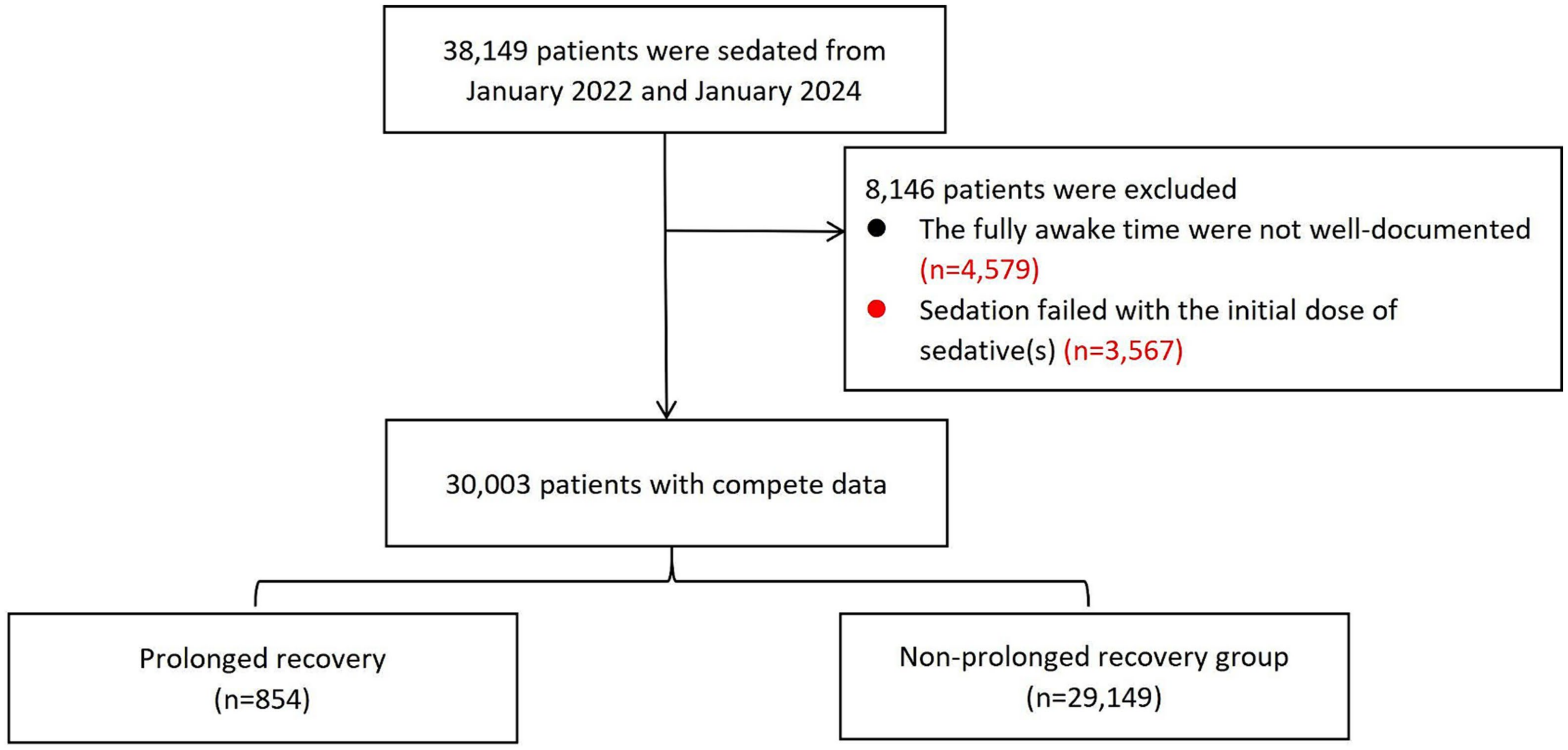
The study flowchart.

A total of 38,149 patients were sedated between January 2022 and January 2024. Of these, 8,146 patients were excluded because the fully awake time was not well-documented or sedation failed with the initial sedative(s). Finally, 30,003 patients were included in the analysis. The study flow chart was shown in Figure 1.

### Demographics and sedation characteristics in different age groups

The demographic characteristics are summarized in Table 1. The cases were divided into 4 age groups: neonates (≤ 28 days) with 749 cases, infants (29 days-1 year old) with 12,266 cases, toddlers (1–3 years old) with 10,857 cases, and old children (>3 years old) with 6,131 cases. Among the cases, 60.4% were boys. The weight of enrolled patients was 10.0 kg (IQR 7.5–13.5 kg). The most common diagnostic examination was MRI (9,163, 30.5%), followed by lung function test (7,972, 26.6%), hear screening (4,369, 14.6%), and echocardiography (4,242, 14.1%). Most patients received chloral hydrate as the initial sedative (17,538, 58.6%), followed by midazolam combined dexmedetomidine (9,534, 31.8%), chloral hydrate combined dexmedetomidine (2,515, 8.4%), and others (54, 0.2%). The median duration of sedation was 58.2 min (IQR 47.0–73.1 min).

**Table 1:** Characteristics of patients undergoing procedural sedation, grouped by age.

Variables	All (*n* = 30003)	Neonates (*n* = 749)	Infants (*n* = 12266)	Toddlers (*n* = 10857)	Old children (*n* = 6131)
Gender, males, *n* (%)	18125 (60.4)	442 (59.0)	7210 (58.8)	6538 (60.2)	3935 (64.2)
Weight, Kg, Median(IQR)	10.0 (7.5, 13.5)	3.3 (2.9, 3.7)	7.1 (5.9, 8.5)	12.0 (10.5, 13.0)	15.5 (14.0, 18.0)
Type of patients, *n* (%)					
Outpatients	19441 (64.8)	156 (20.8)	8387 (68.4)	6866 (63.2)	4032 (65.8)
Inpatients	10562 (35.2)	593 (79.2)	3879 (31.6)	3991 (36.8)	2099 (34.2)
Sedation history (Yes), *n* (%)	10184 (33.9)	135 (18.0)	3239 (26.4)	4328 (39.9)	2482 (40.5)
Sleep deprivation (Yes), *n* (%)	7314 (24.4)	177 (23.6)	2507 (20.4)	2689 (24.8)	1941 (31.7)
Type of procedures, *n* (%)					
VAEP	540 (1.8)	26 (3.5)	224 (1.8)	108 (1.0)	182 (3.0)
Echocardiography	4242 (14.1)	3 (0.4)	2741 (22.4)	1469 (13.5)	29 (0.5)
CT	2747 (9.2)	15 (2.0)	928 (7.6)	1326 (12.2)	478 (7.8)
Hear screening	4369 (14.6)	1 (0.1)	3199 (26.1)	806 (7.4)	363 (5.9)
Lung function test	7972 (26.6)	2 (0.3)	2154 (17.6)	4273 (39.4)	1543 (25.2)
MRI	9163 (30.5)	593 (79.2)	2689 (21.9)	2503 (23.1)	3378 (55.1)
Others	18 (0.1)	1 (0.1)	4(0.0)	10 (0.1)	3 (0.1)
More than one procedure†	952 (3.2)	108 (14.4)	327 (2.7)	362 (3.3)	155 (2.5)
Drugs used, *n* (%)					
Chloral hydrate	17538 (58.5)	726 (96.9)	11623 (94.8)	3877 (35.8)	1312 (21.5)
Chloral hydrate+Dexmeditomidine	2515 (8.4)	1 (0.1)	109 (0.9)	1036 (9.6)	1369 (22.4)
Chloral hydrate+Midazolam	52 (0.2)	1 (0.1)	10 (0.1)	13 (0.1)	28 (0.5)
Midazolam	310 (1.0)	17 (2.3)	23 (0.2)	158 (53.1)	112 (1.8)
Midazolam+Dexmeditomidine	9534 (31.8)	0 (0.0)	496 (4.1)	5748 (53.1)	3290 (52.8)
Others	54 (0.2)	4 (0.5)	5 (0.0)	25 (0.2)	20 (0.3)
Sedation recovery, min, Median (IQR)	58.2 (47.0, 73.1)	54.1 (43.5, 70.0)	56.0 (45.0, 71.0)	61.0 (48.2, 76.0)	60.0 (49.0, 73.0)
Prolonged sedation recovery, *n* (%)	854 (2.8)	12 (1.6)	417 (3.4)	267 (2.5)	158 (2.6)

VAEP visual and auditory evoked potential, CT computer tomography, MRI magnetic resonance imaging.

^†Patients underwent two procedures simultaneously.^

**Figure 2:**
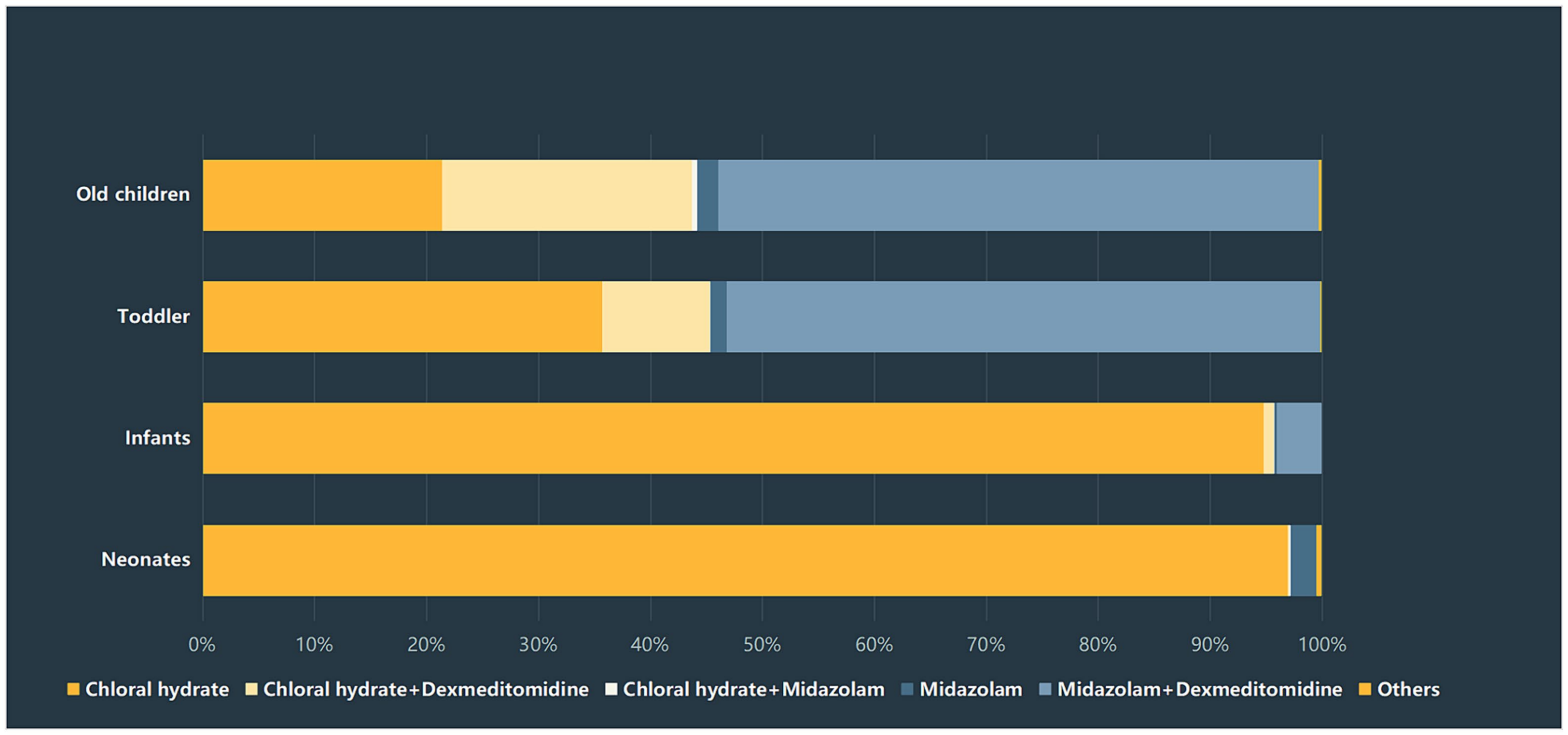
Sedatives selection among different age groups.

The duration of sedation recovery was compared among the four age groups, and significant differences were detected among the four groups (*p* < 0.01). The highest rate of prolonged sedation recovery was found in the infants group (3.4%), followed by the old children group (2.6%) and the toddlers group (2.5%). The neonates group had the lowest rate of prolonged recovery, which was 1.6%. There was a significant difference in the rate of prolonged recovery between the infants group and the other three groups. Notably, the selection of sedatives varied greatly among the four age groups. More than 90% of neonates and infants were administered chloral hydrate, while only 35.8% of infants and 21.5% of old children received chloral hydrate, respectively. In contrast, about 53.1% of toddlers and 52.8% of old children chose midazolam + dexmedetomidine for procedural sedation. The distribution of sedatives selection among the four groups was presented in Figure 2.

### The incidence of prolonged sedation recovery

The incidence of prolonged sedation recovery time with the initial drugs and its underlying cause Among the enrolled 30,003 patients, 854 subjects (2.8%) experienced prolonged recovery. The weight of patients was 9.5 (6.0, 13.5) kg in the PSR group and 10.0 (7.5, 13.5) kg in the NPSR group, with a statistical significance (*p* < 0.05).

### Risk factors for prolonged recovery by univariate analysis and LASSO regression analyses

As shown in Table 2, the univariate analysis indicated that neonates, infants, toddlers, body weight, type of patients, patients with sedation history, patients undergoing VAEP, echocardiography, CT, hear screening, lung function test, MRI, more than one procedures simultaneously, patients received chloral hydrate, patients received chloral hydrate + dexmedetomidine, and patients received midazolam + dexmedetomidine were associated with the prolonged sedation recovery with the initial sedative(s).

**Table 2:** Univariate and multivariate logistic analysis of risk factors associated with prolonged recovery.

	Univariable logistic regression			
	PSR group (*n* = 854)	NPSR group (*n* = 29149)	OR (95% CI)	*p* values
Gender, Male, *n* (%)	508 (59.5)	17617 (60.4)	0.96 (0.84, 1.10)	0.58
Age, *n* (%)				
Neonates (≤28 days)	12 (1.4)	737 (2.5)	0.55 (0.31, 0.98)	0.04^*^
Infants (28 days ~ 1 year)	417 (48.8)	11849 (40.7)	1.39 (1.22, 1.60)	<0.01^*^
Toddlers (1 year ~ 3 years)	267 (31.3)	10590 (36.3)	0.8 (0.69, 0.92)	<0.01^*^
Old children (>3 year)	158 (18.5)	5973 (20.5)	0.88 (0.74, 1.05)	0.16
Weight (Kg), Median (IQR)	9.5 (6.0, 13.5)	10.5 (7.5, 13.5)	0.98 (0.96, 0.99)	<0.01^*^
Type of patients, Outpatients, *n* (%)	707 (82.8)	18734 (64.3)	2.67 (2.23,3.2)	<0.01^*^
Sedation history, Yes, *n* (%)	362 (42.4)	9822 (33.7)	1.45 (1.26, 1.66)	<0.01^*^
Sleep deprivation, Yes, *n* (%)	218 (25.5)	7096 (24.3)	1.07 (0.91, 1.24)	0.43
Type of procedures, *n* (%)				
VAEP	6 (0.7)	534 (1.8)	0.38 (0.17, 0.85)	0.02^*^
Echocardiography	63 (7.4)	4179 (14.3)	0.48 (0.37, 0.62)	<0.01^*^
CT	31 (3.6)	2716 (9.3)	0.37 (0.36, 0.53)	<0.01^*^
Hear screening	506 (59.3)	3863 (13.3)	9.52 (8.27, 10.95)	<0.01^*^
Lung function test	99 (11.6)	7873 (27.0)	0.35 (0.29,0.44)	<0.01^*^
MRI	96 (11.2)	9067 (31.1)	0.28 (0.23, 0.35)	<0.01^*^
Others	1 (0.12)	17 (0.06)	2.01 (0.267, 15.11)	0.50
More than one procedures simultaneously^†^	52 (6.1)	900 (3.1)	2.04 (1.53, 2.71)	<0.01^*^
Type of sedatives, *n* (%)				
Chloral hydrate	687 (80.4)	16851 (57.8)	3.0 (2.53, 3.56)	<0.01^*^
Chloral hydrate+Dexmeditomidine	43 (5.0)	2472 (8.5)	0.57 (0.42, 0.78)	<0.01^*^
Chloral hydrate+Midazolam	2 (0.2)	50 (0.2)	1.37 (0.33, 5.62)	0.67
Midazolam	3 (0.4)	307 (1.1%)	0.33 (0.11, 1.03)	0.06
Midazolam+Dexmeditomidine	117 (13.7)	9417 (32.3)	0.33 (0.27, 0.40)	<0.01^*^
Others	2 (0.2)	52 (0.2)	1.31 (0.32, 5.4)	0.71

VAEP visual and auditory evoked potential, CT computer tomography, MRI magnetic resonance imaging.

^†^Patients underwent two procedures simultaneously. *Bold value: *p* < 0.05.

LASSO regression analyses were conducted to select the optimal variables related to the outcome. When the lambda value was selected as Lambda_1se = 0.002380073, 11 variables with non-zero coefficients were identified, and the predictive variables were presented in Table 3. Then, a predictive model was developed using LASSO regression analysis. Finally, in the adjusted model, a lower body weight (OR 1.03, 95%CI 1.01–1.05), outpatients (OR 1.31, 95%CI 1.07–1.59), patients with sedation history (OR 1.25, 95%CI 1.07–1.44), and patients received chloral hydrate (OR 1.47, 95%CI 1.06–2.03), were associated with increased odds of the prolonged recovery with the initial sedative(s). Infants (OR 0.74, 95%CI 0.61–0.90), patients undergoing VAEP (OR 0.21, 95%CI 0.05–0.26), echocardiography (OR 0.15, 95%CI 0.12–0.20), CT (OR 0.12, 95%CI 0.08–0.17), lung function test (OR 0.13, 95%CI 0.10–0.17), and MRI (OR 0.09, 95%CI 0.07–0.12), were associated with reduced odds of the prolonged recovery after sedation (Table 3). None of the variables in the final model had collinearity problems.

**Table 3:** LASSO regression analysis of factors associated with prolonged recovery

	LASSO logistic regression	
	Adjusted OR (95% CI)	*p* (Wald’s test) values
Infants (28 days ~ 1 year)	0.74 (0.61, 0.90)	<0.01^*^
Weight (Kg), Median (IQR)	1.03 (1.01, 1.05)	<0.01^*^
Type of patients, Outpatients, *n* (%)	1.31 (1.07, 1.59)	<0.01^*^
Sedation history, Yes, *n* (%)	1.25 (1.07, 1.44)	<0.01^*^
Type of procedures, *n* (%)		
VAEP	0.12 (0.05, 0.26)	<0.01^*^
Echocardiography	0.15 (0.12, 0.20)	<0.01^*^
CT	0.12 (0.08, 0.17)	<0.01^*^
Lung function test	0.13 (0.10, 0.17)	<0.01^*^
MRI	0.09 (0.07, 0.12)	<0.01^*^
Type of sedatives, *n* (%)		
Chloral hydrate	1.47 (1.06, 2.03)	0.02^*^

VAEP visual and auditory evoked potential, CT computer tomography, MRI magnetic resonance imaging.

^†^Patients underwent two procedures simultaneously. *Bold value: *p* < 0.05.

## Discussion

In the current study, a total of 30,003 children were analyzed. Of those, 854 patients developed prolonged recovery with the initial drugs. The incidence of prolonged recovery during procedural sedation was 2.8%. Different age groups presented varying sedation characteristics. In the adjusted model, a higher body weight, outpatients, patients with a sedation history, and patients who received chloral hydrate were associated with increased odds of prolonged recovery with the initial sedative(s). However, infants, and patients undergoing VAEP, echocardiography, CT, lung function test, and MRI were less likely to develop prolonged recovery after sedation.

In our study, we found that the sedation medication regimens of the four age groups of children varied considerably. The initial dose and drug types prescribed to a patient depend on patient-related factors, the depth of sedation required for different procedures, and providers education and experience. In our center, chloral hydrate was the most commonly used drug during non-invasive diagnostic procedures until 2022; whereas, after 2022, dexmedetomidine and midazolam were used increasingly. We assumed that at the initial stage, some providers still prefer to use chloral hydrate for procedural sedation due to the extensive experience of this medication.

### The incidence of prolonged sedation recovery

The reasons for a prolonged recovery after sedation are complex and influenced by patients age, obesity, medical history, medication used, and other factors (1–3, 11). Our study found that about 2.8% of children experienced sedation-related prolonged recovery, which was somewhat lower than was reported by Zhou et al. (3). This discrepancy might be due to variations in the type and dose selection of sedatives. First, in our institution, the sedative medicines were selected at the providers’ discretion. The results showed that chloral hydrate, chloral hydrate combined dexmedetomidine, chloral hydrate combined midazolam, midazolam, and midazolam combined dexmedetomidine, were prescribed for procedural sedation. In recent years, we have explored several different sedation regimens to improve sedation quality while minimizing adverse reactions (9, 12). Second, we reported that dexmedetomidine was initially prescribed at a relatively lower dose (≤1.0 mcg/kg) in our sedation center, in combination with oral midazolam to improve sedation success and decrease safety concerns (9). However, Zhou et al. administrated 2 mcg/kg intranasal dexmedetomidine and 0.5 mg/kg oral midazolam (3). Obviously, they gave a higher dose than us. Theoretically, a higher dose could lead to a longer sedation effect. The previous study supported that intranasal administration at 2 μg/kg dexmedetomidine could achieve a target sedation level that lasted for up to 2 h (13). Last, in our study, about 35.2% of patients were hospitalized, which might be related to early discharge from the sedation center to return to the ward for monitoring and further treatment.

### Factors associated with increased odds of prolonged recovery

Similar to the results of the previous publications (2, 14, 15), we found that a heavier body weight predicted the risk of prolonged recovery. Drug absorption, distribution, and metabolism are all affected by an increase in BMI (16). Most sedative drugs are lipophilic, which results in free active drugs, being present in high concentrations in fat tissue (14). Patients with a heavier weight have a greater volume of distribution for lipophilic drugs. Thus, the clinical duration of these drugs is likely to be extended (17). Besides, oral administration of sedative drugs was usually dosed by actual body weight rather than the predicted body weight. Dosing drugs to actual body weight might result in over-dose and likely excessive effects.

Outpatients had been linked to prolonged recovery. The definition of prolonged recovery was the period between administration of sedative(s) until patients discharged from sedation center. The possible explanation is that the discharge determination for outpatients will be more cautious compared to inpatients. Inpatients may be continuously monitored in the ward, even after they have been discharged from the sedation center. However, it is impossible for outpatients. To ensure safety, outpatients were preferred to be monitored for a longer period of time by the sedation providers.

Patients with sedation history were also identified as the risk factors associated with prolonged recovery after sedation. The underlying mechanism of this effect is unknown. However, in our previous work, we found that patients with a history of sedation with an increased odds of sedation failure at the initial dose (7). We assumed that according to our previous results, the sedation providers might choose to give a higher dose of sedatives for those patients. The duration of sedation is proportional to the dose of sedative drugs. Besides, patients with a sedation history might be a marker of increased age. Older children were known to be more difficult to sedate than infants (18). This is due to changes in their cognition.

Another sedative-related risk factor identified in the current study was chloral hydrate administration, which had been supported by previous publications (4, 19). A systematic review including six randomized clinical trials demonstrated that compared to intranasal dexmedetomidine, oral chloral hydrate took a longer time to return to baseline physical activity on the same day of the procedure (19). Compared with oral chloral hydrate, intranasal dexmedetomidine has a shorter wake-up time (WMD = −9.75, 95% CI: −17.57 to −1.94, *p* = 0.014) (20). In Italy, chloral hydrate was not encouraged to be used for sedation and it was not commercially available since 2016 due to its side effects, such as prolonged sedation, hyperactivity, and nervousness (4, 21). However, the guidelines proposed in 2024 for diagnostic procedural sedation in South Korea recommended that oral chloral hydrate could be used based on the patient’s condition and availability of the medication (22). To our knowledge, no consensus was achieved on the optimal sedative drugs. The choice of sedation drugs is not only depended on the procedure itself and on the comorbidities of the child, but also on the experience of the sedation provider, degree of sedation-related training, and drug availability. Thus, it is particularly pivotal to seek a sedative drug with higher safety and efficacy to assist procedural sedation based on certain circumstances.

### Factors associated with reduced odds of prolonged recovery

The previous study showed that infants had a longer sedation time and a higher rate of delayed recovery after procedural sedation (3). Thus, we also analyzed the patients according to their age. However, in the final model, we found that infants were less likely to experience prolonged sedation recovery, which contradicts the previous conclusions (3). This discrepancy might be attributed to differences in the definition of infants. In our study, we further divided pediatric patients (aged<1 year old) into the neonatal group and the infant group. According to our results, the neonates group had the lowest rate of prolonged recovery, which was 1.6%. However, most neonates were inpatients from the neonatal intensive care unit (NICU). To our knowledge, the NICU has sufficient monitoring and management capabilities for emergencies, while the beds in the recovery room are limited. To improve bed turnover, those groups of patients might be transferred back to the NICU early for monitoring and further treatments, even if they were not fully awake. Moreover, some immobilization devices and noise-canceling headphones were routinely used in neonates, which allowed patients to receive lower doses of sedatives and the neonates could be slightly awake. Those accounted for a lower rate of prolonged recovery. Although the highest rate of prolonged recovery after sedation was found in the infants group, we thought there was some bias. Table 1 showed that 94.8% of them were administrated chloral hydrate. As aforementioned, chloral hydrate was a sedative-related risk factor for prolonged recovery, which was a potential confounding factor on the results about the group of infants that had a higher rate of prolonged recovery. We assumed that the results might be changed when this factor was adjusted. As expected, after performing multivariable logistic regression and adjusting the odds ratio (OR), infants became a factor associated with reducing the odds of prolonged recovery.

### Limitations

There were several limitations. First, although the study enrolled 30,003, all the data was collected from a single center, leading to the fact that these results were only empirical to that institution’s specific sedation practices and outcomes. The ability to generalize the experiences to other centers is therefore questionable. Sedation practices might vary across different healthcare facilities, depending on drug availability, providers’ education and experience, and internal policy. Next, due to busy office-based practice, objective sedation scoring tool, such as Aldrete or Rasmay score, did not routinely assessed and recorded. However, we defined that the patients achieved a satisfied depth of sedation when they could complete the procedures without rescue medications, which was also reasonable during the clinical practice. We thought this might be a practice-based substitution for sedation score, especially in a high-volume center. Last, the study did not account for potential confounding factors that could influence sedation outcomes, such as pre-procedural fasting times, sedative medication dose, underlying medical conditions, concurrent medications, or procedural complexity. Unfortunately, not all confounding factors were documented in the medical records since this was a retrospective study and most patients were outpatients. Moreover, sedation duration was affected by multiple factors. For example, when a patient has completed the procedure, they may be artificially awakened from sedation by the staff, especially near the end of the day. Thus, sedation duration might be influenced by the extra stimulation (23). However, we were unable to distinguish if the child woke up naturally or by external stimuli as this confounding factor was not recorded.

## Conclusion

In the current study, we found that the incidence of prolonged recovery was 2.8%. A higher body weight, outpatients, patients with sedation history, and patients received chloral hydrate were associated with increased odds of the prolonged recovery with the initial sedative(s). For patients with these characteristics, the monitoring time needs to be extended.

## Data Availability

The raw data supporting the conclusions of this article will be made available by the authors, without undue reservation.
